# The Role of −786T/C Polymorphism in the Endothelial Nitric Oxide Synthase Gene in Males with Clinical and Biochemical Features of the Metabolic Syndrome

**DOI:** 10.1155/2011/458750

**Published:** 2011-11-24

**Authors:** Blazej Misiak, Marta Krolik, Anna Kukowka, Anna Lewera, Przemyslaw Leszczynski, Joanna Stankiewicz-Olczyk, Ryszard Slezak

**Affiliations:** ^1^Department of Genetics, Wroclaw Medical University, 50-368 Wroclaw, Poland; ^2^Copper Health Centre, The Outpatient Clinic of Endocrinology, 59-300 Lubin, Poland

## Abstract

*Background*. Extensive evidence, arising from models of endothelial nitric oxide synthase gene (*NOS3*)-knockout mice supports the role of endothelial malfunction in the pathogenesis of the metabolic syndrome (MS). *Aims*. The aim of this study was to evaluate the role of −786T/C polymorphism in the etiology of MS and assess previously reported interaction with cigarette smoking. *Methods*. Based on International Diabetes Federation 2005 criteria, we recruited randomly 152 subjects with MS and 75 subjects without MS. *Results*. Allelic and genotype frequencies did not differ significantly between both groups. Total cholesterol level (CHOLT) and intima-media thickness of carotid arteries were significantly higher in −786CC homozygotes, in comparison with −786TC and −786TT patients. Regarding current smoking status, −786C allele was associated with higher CHOLT than −786T allele. *Conclusion*. Our study indicates the putative role of −786T/C polymorphism in the development of hypercholesterolemia, in patients with MS, which might be enhanced by cigarette smoking.

## 1. Introduction

The metabolic syndrome (MS) is a clustering entity, characterized by obesity, hypertension, hyperglycemia, dyslipidemia, and insulin resistance. Definitions of MS were created in order to indicate the patients at increased risk of cardiovascular complications and type 2 diabetes. Currently recommended criteria for MS diagnosing were proposed by National Cholesterol Education Program (NCEP), Adult Treatment Panel III (ATP III), and International Diabetes Federation 2005 (IDF 2005). 

Atherosclerotic lesions may be the complication of MS, arising from endothelial dysfunction and induced by decreased nitric oxide (NO) production. NO is synthesized by nitric oxide synthase (eNOS), encoded by *NOS3* gene, and displays anti-inflammatory, vasodilatory, and antiproliferative effects [1]. Recent studies also demonstrate that *NOS3*-knockout mice develop dyslipidemia, insulin resistance, decreased insulin-dependent glucose uptake in skeletal muscles and adipose tissue, and hypertension, mimicking the phenotype of MS [2–5]. In humans *NOS3* gene is located at 7q35-36 and possesses a few polymorphic variants. Several reports indicate the potential association between *NOS3* gene polymorphisms and MS [6–8]. Functional polymorphism in the promoter region (−786T/C) may alter *NOS3* gene expression [9, 10] and lead to decreased biosynthesis of NO [11]. Although the impact of −786T/C polymorphism seems to be well established with regard to several cardiovascular end points, its exact role in the etiology of MS still remains unclear.

Besides genetic risk factors, atherosclerotic vessel damage may occur due to modifiable, environmental insults. Moreover, a growing body of evidence suggests the role of gene × environment interactions (G × E) in the etiology of multifactorial diseases including MS, type 2 diabetes, and atherosclerosis [12]. Following this notion, cigarette smoking may directly reduce eNOS activity and enhance the endothelial malfunction. However, this alteration probably depends upon *NOS3* genotype [13]. 

The aim of this study was to evaluate the association between −786T/C polymorphism and MS, and to assess the putative G × E interaction with cigarette smoking. 

## 2. Materials and Methods

### 2.1. Subjects

We recruited randomly 227 males without any previous treatment of dyslipidemia, diabetes, or cardiovascular diseases. To avoid the potential hormonal influences and assess the notion that gender may be the risk factor for vascular incidents, we limited our study to male individuals. All subjects were Caucasians, worked in the copper smelter, and came from the same geographic area. Written informed consent was obtained from all volunteers after the approval of local Ethics Committee. 

Anthropometric measurements were carried out by trained medical staff and included weight, height, waist, and hip circumference. On the basis of anthropometric measurements, waist-to-hip ratio (WHR) and body mass index (BMI) were calculated. Hypertension was diagnosed based on blood pressure measurements in a quiet room after lying down for 15 minutes, which were performed at least twice during two or three different appointments. Diabetes type 2 or impaired fasting glucose were diagnosed using WHO criteria. Triglycerides (TG), total cholesterol (CHOLT), low-density lipoproteins (LDL) and high-density lipoproteins (HDL) levels were estimated according to standard protocols. The compound panel of biochemical measurements was performed in all subjects from both groups and included coagulation parameters (international normalized ratio, prothrombin index, and fibrinogen concentration), hormones concentrations (thyrotropin, testosterone, insulin), C-reactive protein (CRP), uric acid (UA), and leptin and adiponectin concentrations. 

Subjects, who fulfilled IDF 2005 criteria for MS, were recruited into the study group (152 males) and others, without clinical and biochemical features of MS were enrolled into the control group (75 males).

### 2.2. DNA Extraction and Genotype Analysis

Genetic analysis was performed according to the PCR-RFLP protocol, described by Imamura et al. [14] with the following oligonucleotide primers: 5′-CAT TCT GGG AAC TGT-3′ as a forward sequence and 5′-GTC AGC AGA GAG ACT-3′ as a reverse sequence. PCR product had been digested using *MspI* restriction endonuclease and visualized on 2.5% agarose gel.

### 2.3. Statistics

Statistical analysis has been performed using STATISTICA v 9.0 software. Chi-square test has been used for the evaluation of genetic linkage between −786T/C polymorphism and MS. The mean values of clinical parameters with regard to −786T/C polymorphism have been analyzed in the Kruskal-Wallis test. Baseline characteristics and the influence of cigarette smoking on CHOLT and IMT have been assessed using the Mann-Whitney *U* test.

## 3. Results

Characteristics of study and control group are shown in Table 1. Genotype and allelic frequencies are presented in Table 2. The difference in −786T/C polymorphism distribution between MS patients and healthy subjects was not significant. 

Analysis of mean clinical parameters with regard to −786T/C polymorphism in study group revealed significant results (Table 3). The difference in CHOLT between MS subjects with distinct −786T/C genotypes was statistically significant (−786TT : 5.44 mmol/L, −786TC : 5.47 mmol/L, and −786CC : 5.78 mmol/L; *P* = 0.04). Similarly, both left and right IMT were significantly lower in −786TT homozygotes, in comparison with −786TC and −786CC patients (for left IMT : 0.56 cm, 0.57 cm, and 0.65 cm resp., *P* = 0.04 and for right IMT: 0.54 cm, 0.58 cm, and 0.65 cm resp., *P* = 0.02). 

The interaction between cigarette smoking and studied polymorphism was assessed in both groups simultaneously and revealed significant results regarding only CHOLT (Table 4). The prevalence of current cigarette smoking was 59.2% (90 subjects) in study group and 62.6% (47 subjects) in control group. CHOLT was significantly higher (*P* = 0.03) in −786TC smokers (5.74 mmol/L) in comparison with −786TC nonsmokers (5.3 mmol/L). Higher CHOLT (*P* = 0.01) was also found in −786CC smokers (5.66 mmo/L) than in −786CC nonsmokers (4,85 mmol/L). The difference in CHOLT between −786TT smokers and −786TT nonsmokers was not statistically significant (5.28 mmol/L versus 5.08 mmol/L, *P* = 0.47). Regarding allele carrier status, −786T allele was associated with significantly lower (*P* = 0.003) CHOLT with respect to −786C allele (5.23 mmol/L versus 5.73 mmol/L). It is noteworthy that the mean values of CHOLT did not differ significantly between subjects with distinct genotypes either in smokers or nonsmokers (data not shown).

## 4. Discussion

Our study did not reveal significant association of −786T/C polymorphism with MS. The subthreshold results may arise from the study design limitations, originating from the sample size. However, interesting results were obtained concerning clinical parameters of MS. We confirmed the putative impact of −786T/C polymorphism on atherosclerotic lesions by measuring IMT in carotid arteries. These findings are consistent with previously published data, based on various imaging indices of atherosclerosis in MS patients including aortic and peripheral wave velocity [15] or ankle-brachial index [16]. According to previously published data, derived from transcriptional studies, it is unexplained clearly whether −786T/C polymorphism may decrease the expression of *NOS3* gene and lead to reduction in NO release [9, 17, 18]. Miyamoto et al. [19] have found the potential mechanism underlying this phenomenon. They have observed that only −786C variant is targeted by replication protein A1, which is a single-strand DNA-binding transcription factor repressing transcription of *NOS3 in vitro*.

The novel finding, resulting from our study, concerns the influence of −786T/C on CHOLT, which was significantly higher in −786CC homozygotes, in comparison with other genotypes. Emerging data indicate that *NOS3*-knockout mice display hypercholesterolemia among other clinical traits of MS [5, 20]. Although the direct connection between NO activity and lipid metabolism remains unclear, we hypothesize that the impact of −786T/C polymorphism in *NOS3 *gene on clinical features of MS including cholesterol metabolism might be less severe than the knockout of the whole gene. Moreover, the genetic etiology of MS originates from interactions between a plethora of modest effect loci, which impair different clinical parameters of this clustering entity. Hence, the only clinical outcome from this study concerns alterations in CHOLT and IMT. 

Furthermore, we evaluated the possible G × E interactions by assessing the synergism between cigarette smoking and −786T/C polymorphism. According to our results, the influence of −786T/C polymorphism might be enhanced by tobacco smoke ingredients. Statistical analysis revealed significant correlations between CHOLT and −786T/C polymorphism in conjunction with current smoking status. The interaction between smoking and *NOS3* polymorphisms has been assessed in several studies with regard to coronary artery disease [21, 22], peripheral arterial disease [16], and cerebrovascular incidents [23]. Moreover, cigarette smoking may in itself affect NO release [24, 25]. According to the current knowledge, exposure to cigarette smoke ingredients may impair eNOS activity in various mechanisms including release of reactive oxygen species, which contribute to eNOS uncoupling [26], targeting *NOS3* gene expression [27, 28], altering phosphorylation of eNOS via interactions with protein kinase C [29], as well as accelerating catabolism of tetrahydrobiopterin, which is the eNOS-cofactor [30]. The limitation of this study is that we did not assess the putative dose-dependent effect of active smoking. However, previous studies suggest that heavy and light smokers may display similar deleterious effects on NO biosynthesis [31].

## 5. Conclusions

Our results support the influence of −786T/C polymorphism on clinical features of MS. Moreover, this study sheds new light on the role of NO in the development of hypercholesterolemia. Studied polymorphism may alter *NOS3* gene expression and contribute to lipid dysmetabolism. Thus, observed impact of −786T/C polymorphism on atherosclerotic indices not only might constitute the direct effect of endothelial damage but could also appear due to elevated CHOLT. On the other hand, −786C allele may enhance deleterious effects of cigarette smoking. However, the exact mechanism of this G × E interaction requires further investigation.

## Figures and Tables

**Table 1:** Baseline characteristics of study group and control group.

Parameter	Study group	Control group	*P*-value*
Age	46,35 ± 6,64	43,22 ± 5,84	0,001
BMI (kg/m^2^)	30,49 ± 2,91	24,46 ± 2,08	<0,001
Fatty tissue percentage (%)	28,81 ± 5,72	18,53 ± 4,37	<0,001
Waist circumference (cm)	102,09 ± 7,81	86,79 ± 4,80	<0,001
Hip circumference (cm)	106,75 ± 5,75	98,8 ± 3,51	<0,001
WHR	0,96 ± 0,04	0,88 ± 0,03	<0,001
CHOLT (mmol/L)	5,64 ± 0,93	5,01 ± 0,79	<0,001
LDL (mmol/L)	3,44 ± 0,89	2,84 ± 0,72	<0,001
HDL (mmol/L)	1,31 ± 0,29	1,64 ± 0,42	<0,001
TG (mmol/L)	2,30 ± 1,32	1,18 ± 0,45	<0,001
Uric acid (*μ*mol/L)	368,08 ± 67,13	306,48 ± 61,91	<0,001
CRP (mg/L)	2,89 ± 2,96	1,08 ± 0,92	<0,001
Leptine (ng/mL)	9,59 ± 5,59	4,61 ± 2,04	<0,001
Adiponectine (*μ*g/mL)	6,95 ± 3,50	8,94 ± 3,16	0,002
Left IMT (cm)	0,59 ± 0,15	0,51 ± 0,09	<0,001
Right IMT (cm)	0,59 ± 0,17	0,50 ± 0,08	<0,001
HOMA-IR	3,45 ± 2,21	1,37 ± 0,69	<0,001
QUICKI	0,68 ± 0,41	0,78 ± 0,50	0,18

Results expressed as mean values ± SD. Abbreviations: BMI: body mass index, WHR: waist-to-hip ratio, CHOLT: total cholesterol level, LDL: low density lipoproteins, HDL: high density lipoproteins, TG: trigllcerides, CRP: C-reactive protein, IMT: intima-media thickness of carotid arteries, HOMA-IR: Homeostasis Model of Assessment-Insulin Resistance, QUICKI: Quantitative Insulin-Sensitivity Check Index.

*P-value refers to the Mann-Whitney *U* test.

**Table 2:** Genotype and allelic frequency in study group and control group.

	Study group	Control group	OR	95% CI	*P*-value*
	*N* = 152	*N* = 75			
−786TT −786TC −786CC	41 (27%) 89 (58,5%) 22 (14,5%)	25 (33,3%) 41 (54,7%) 9 (12%)	referent 1,17 1,24	0,65–2,04 0,53–2,93	0,61 0,59
−786T	171 (56,25%)	91 (60,67%)	referent		
−786C	133 (43,75%)	59 (39,33%)	1,19	0,79–1,80	0,37

*P-value calculated in *χ* ^2^ test.

**Table 3:** The comparison of mean values of studied parameters in MS subjects.

Parameter	−786TT	−786TC	−786CC	*P*-value*	−786T	−786C	*P*-value**
Overweight percentage (%)	24.21 ± 11.95	24.14 ± 13.34	25.08 ± 14.9	0.75	24.16 ± 13.02	24.44 ± 13.8	0.98
BMI (kg/m^2^)	30.6 ± 2.97	30.41 ± 2.93	30.63 ± 2.89	0.91	30.45 ± 2.91	30.46 ± 2.93	0.95
Waist circumference (cm)	101.81 ± 7.73	102.36 ± 7.78	102.53 ± 8.16	0.87	101.93 ± 7.71	102.05 ± 7.84	0.95
Hip circumference (cm)	106.05 ± 6.01	107 ± 5.81	107.05 ± 5.14	0.31	106.7 ± 5.87	107.01 ± 5.66	0.69
Fatty tissue percentage (%)	29.15 ± 5.37	28.81 ± 7.11	29.15 ± 5.37	0.87	28.7 ± 5.86	28.82 ± 5.47	0.69
WHR	0.95 ± 0.04	0.96 ± 0.03	0.97 ± 0.06	0.84	0.95 ± 0.04	0.96 ± 0.05	0.71
Prothrombin index (%)	103.02 ± 6.53	100.7 ± 8.48	99.44 ± 8.93	0.35	101.17 ± 8.15	100.3 ± 8.61	0.62
INR	1.25 ± 1.45	0.99 ± 0.08	0.97 ± 0.06	0.31	1.07 ± 0.82	0.99 ± 0.08	0.51
Fibrynogen (g/L)	3.23 ± 0.65	3.41 ± 0.58	3.56 ± 0.48	0.19	3.37 ± 0.59	3.46 ± 0.55	0.46
CHOLT (mmol/L)	5.44 ± 0.85	5.47 ± 0.98	5.78 ± 0.94	**0.04**	5.67 ± 0.92	5.72 ± 0.95	0.58
LDL (mmol/L)	3.30 ± 0.82	3.34 ± 0.82	3.52 ± 0.94	0.11	3.45 ± 0.91	3.49 ± 0.92	0.64
HDL (mmol/L)	1.32 ± 0.27	1.29 ± 0.36	1.28 ± 0.28	0.6	1.31 ± 0.27	1.32 ± 0.29	0.76
TG (mmol/L)	1.83 ± 0.59	2.19 ± 1.2	2.47 ± 1.47	0.19	2.34 ± 1.36	2.37 ± 1.39	0.78
Uric acid (*μ*mol/L)	361.72 ± 69.2	368.09 ± 64.7	381.73 ± 63.26	0.19	362.99 ± 68.09	368.08 ± 67.78	0.52
CRP (mg/L)	2.75 ± 2.12	2.77 ± 2.64	3.36 ± 2.98	0.86	2.91 ± 3.01	2.95 ± 3.22	0.91
TSH (mU/L)	1.52 ± 1.21	1.53 ± 0.75	1.67 ± 0.88	0.21	1.52 ± 1.08	1.55 ± 1.15	0.92
Testosterone (pg/mL)	4.64 ± 1.51	4.1 ± 1.3	4.05 ± 1.4	0.28	4.08 ± 1.33	4.21 ± 1.36	0.54
Leptin (ng/mL)	9.11 ± 4.51	9.61 ± 6.21	10.39 ± 4.85	0.46	9.45 ± 5.71	9.76 ± 5.95	0.69
Adiponectin (*μ*g/mL)	7.28 ± 4.12	7.23 ± 3.54	6.14 ± 2.95	0.23	7.24 ± 3.65	6.88 ± 3.39	0.68
Right IMT (mm)	0.54 ± 0.11	0.58 ± 0.15	0.65 ± 0.23	**0.02**	0.57 ± 0.14	0.6 ± 0.18	0.15
Left IMT (mm)	0.56 ± 0.12	0.57 ± 0.13	0.65 ± 0.2	**0.04**	0.56 ± 0.12	0.59 ± 0.16	0.29
HOMA-IR	3.35 ± 2.27	3.48 ± 1.56	3.63 ± 2.4	0.50	3.37 ± 2.15	3.44 ± 2.31	0.78
QUICKI	0.74 ± 0.5	0.65 ± 0.28	0.55 ± 0.16	0.07	0.71 ± 0.44	0.69 ± 0.45	0.97

Results expressed as mean values ± SD. Abbreviations: BMI: body mass index. WHR: waist-to-hip ratio. INR: International Normalized Ratio. CHOLT: total cholesterol level. LDL: low density lipoproteins. HDL: high density lipoproteins. TG: triglycerides. CRP: C-reactive protein. TSH: thyrotropin. IMT: intima-media thickness of carotid arteries. HOMA-IR: Homeostasis Model of Assessment-Insulin Resistance. QUICKI: Quantitative Insulin-Sensitivity Check Index.

*P-value refers to the Kruskal-Wallis test and **P-value refers to the Mann-Whitney *U* test.

**Table 4:** The influence of cigarette smoking on total cholesterol level (CHOLT) and intima-media thickness (IMT) in men with particular genotype or allele of −786T/C polymorphism.

Genotype/allele	Parameter	Smokers	Non-smokers	*P*-value*
−786CC	CHOLT (mmol/L) Left IMT (cm) Right IMT (cm)	5.66 0.55 0.52	4.85 0.53 0.5	**0.01** 0.81 0.96

−786TC	CHOLT (mmol/L) Left IMT (cm) Right IMT	5.74 0.55 0.56	5.3 0.54 0.55	**0.03** 0.47 0.94

−786TT	CHOLT (mmol/L) Left IMT (cm) Right IMT (cm)	5.28 0.61 0.6	5.08 0.59 0.58	0.47 0.57 0.5

−786C (CC + TC)	CHOLT (mmol/L) Left IMT (cm) Right IMT (cm)	5.73 0.55 0.56	5.27 0.54 0.54	**0.003** 0.41 0.84

−786T (TT + TC)	CHOLT (mmol/L) Left IMT (cm) Right IMT (cm)	5.57 0.56 0.57	5.28 0.55 0.56	0.06 0.61 0.69

Results expressed as mean values. Abbreviations: CHOLT: total cholesterol level. IMT: intima-media thickness of carotid arteries.

*P-value calculated in the Mann-Whitney *U* test.
